# Whole Transcriptome Analysis of Breast Cancer Tumors during Neoadjuvant Chemotherapy: Association with Hematogenous Metastasis

**DOI:** 10.3390/ijms232213906

**Published:** 2022-11-11

**Authors:** Marina K. Ibragimova, Matvey M. Tsyganov, Nikolai V. Litviakov

**Affiliations:** 1 Cancer Research Institute, Tomsk National Research Medical Center of the Russian Academy of Sciences, Tomsk 634009, Russia; 2 Biological Institute, National Research Tomsk State University, Tomsk 634050, Russia; 3 Division of Biochemistry and Molecular Biology with Course of Clinical Laboratory Diagnostics, Siberian State Medical University, Tomsk 634050, Russia

**Keywords:** breast cancer, neoadjuvant chemotherapy, whole transcriptome analysis, tumor expression profile, DEGs, hematogenous metastasis

## Abstract

The assessment of molecular genetic landscape changes during NAC and the relationship between molecular signatures in residual tumors are promising approaches for identifying effective markers of outcome in breast cancer. The majority of the data in the literature present the relationship between the molecular genetic landscape and the response to NAC or are simply descriptive. The present study aimed to determine changes in expression profiles during NAC and assess the relationship between gene expression and the outcome of patients with luminal B HER2 breast cancer depending on distant hematogenous metastasis. The study included 39 patients with luminal B HER2-BC. The patients received 6–8 courses of NAC, and paired samples consisting of biopsy and surgical materials were analyzed. A full transcriptome microarray analysis was performed using the human Clariom™ S Assay platform (Affymetrix, 3450 Central Expy, Santa Clara, CA, 95051, USA). A comparison of the expression profiles of patients with breast cancer before and after NAC, depending on the status of hematogenous metastasis, was conducted. It was shown that the amount of DEGs in the tumor was reduced by more than six times after NAC. The top 10 signaling pathways were also found, the activity of which varied depending on the status of hematogenous metastasis before and after NAC. In addition, the association of DEGs with hematogenous metastasis in patients with breast cancer was evaluated: MFS was assessed depending on the expression level of 21 genes. It was shown that MFS was significantly associated with the expression level and pattern of nine genes. The expression levels of nine DEGs in the tumors of patients with breast cancer after NAC were significantly correlated with MFS when the status of hematogenous metastasis was taken into account.

## 1. Introduction

Predicting the outcome of breast cancer is still a challenging task. It is especially complicated in patients who have undergone neoadjuvant chemotherapy (NAC) since the outcome is strongly correlated with the tumor’s response to NAC [1]. The most significant improvement in long-term results is the observed immediate effect, expressed as complete morphological regression (pCR), the frequency of which is about 18.5% and differs greatly between different IHC subtypes: luminal—8.3–9.3%; triple-negative—31.1–32.6%; and HER2-positive—up to 39–70% [2,3]. According to the RCB (Residual Cancer Burden), with complete morphological regression, the efficiency of predicting a favorable outcome is 90% [4]. The high predictive value of the pathological complete response led the FDA to propose the consideration of pCR as a surrogate marker of long-term outcomes. The effectiveness of RCB (Residual Cancer Burden) as a prognostic marker in patients with residual tumors is 32% and ranges from 22% for HR+/HER2− breast cancers to 44% for HR−/HER2+ breast cancers [4]. In this regard, effective prognostic factors are needed for breast cancer patients with residual tumors after NAC. Patients with the HR+/HER2− molecular subtype are especially in need of such factors since the outcome of the disease can only be effectively predicted in 8.3–9.3% of those who have reached pCR.

Moreover, accurate prediction of recurrence pre- and/or post-NACT through the integration of imaging markers and clinicopathological variables may help improve clinical decision-making in adjusting NACT and/or adjuvant treatment regimens to reduce the risk of recurrence and improve survival outcomes [5].

There is no doubt that NAC greatly changes the primary tumor from stage to stage [6] and in terms of the molecular subtype [7] and molecular genetic landscape [8,9,10]. Changes in the molecular genetic landscape during NAC and the assessment of the relationship between molecular signatures in residual tumors seem to be promising in terms of identifying effective markers for breast cancer outcomes. However, most studies are devoted to the association between the molecular genetic landscape and the response to NAC or are simply descriptive, such as the study published by Loibl, S., [11] who used next-generation sequencing on a large sample (851 patients with breast cancer) to assess genomic changes during preoperative treatment. The mutations of 16 genes—*AKT1*, *BRAF*, *CDH1*, *EGFR*, *ERBB2*, *ESR1*, *FBXW7*, *FGFR2*, *HRAS*, *KRAS*, *NRAS*, *SF3B1*, *TP53*, *HNF1A*, *PIK3CA*, and *PTEN*—were analyzed and changes in the copy number of the genes *CCND*, *ERBB2*, *FGFR1*, *PAK1*, *PIK3CA*, *TOP2A*, *TP53*, and *ZNF703* were estimated. It was revealed that during the process of NAC, the most frequently observed mutations were those of the genes *TP53* (38.4%) and *PIK3CA* (21.5%), and gene amplifications were also present in *TOP2A*, *ERBB2*, and *ZNF703* in more than 30% of cases; *TP53* and *PIK3CA* in more than 20% of cases; and *CCND1*, *PAK1*, and *FGFR* in more than 12% of cases [12]. In another small study of residual tumors in 32 patients with HER2+ breast cancer, it was shown that after NAC, 69% of patients had an increased number of mutated genes (21 gene panels studied) compared to their biopsy; this was shown to be a negative prognostic factor in terms of disease-free survival, as the recurrence rate in the subgroup with a different gene profile was 42% versus 0% in the subgroup with the same profile (*p* = 0.019) [11].

Data regarding the breast tumor transcriptome during preoperative chemotherapy have also been published. In the work of Mark Jesus M. Magbanua et al., the expressions of more than 20,000 genes were analyzed in 36 patients with breast cancer at three points—before treatment (T1), between 24 and 96 h after the first dose of chemotherapy (T2), and in residual tumors after surgery (TS). The results revealed that the expressions of 124 genes (e.g., *GSTP1*, *POU5F1*, *CCRL2*) were significantly modified, and most of the genes were underexpressed at T2. A bioinformatics analysis showed the presence of a group of genes involved in the cell cycle (*n* = 21) as well as a group with roles in cell death and survival (*n* = 38). These included genes encoding kinases such as AURKA and PLK1, which play a key role in cell proliferation. Increased interferon signaling (TS-T1) and the high expression of cell proliferation genes in residual tumors (TS) were of prognostic significance and were associated with reduced RFS [13].

PURPOSE: conducting a whole transcriptome study of a breast tumor of the luminal B HER2− subtype before and after neoadjuvant chemotherapy to determine changes in the expression profile during preoperative treatment and the relationship between gene expression and outcome depending on distant hematogenous metastases (observation period of five years).

## 2. Results

### 2.1. Comparison of the Expression Profile of Patients with Breast Cancer before and after Preoperative Chemotherapy

**Figure 1:**
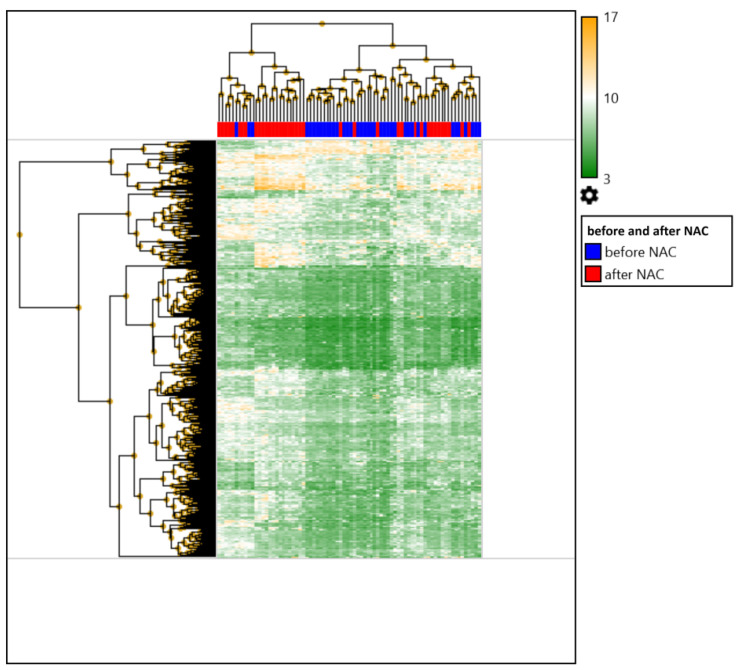
Heat map of DEG in tumors of BC patients before and after NAC. Clustering of data before treatment is shown in blue: by patients (upper side of the heat map), by DEGs (left side of the heat map), clustering after NAC is shown in red: by patients (upper side of the heat map), by DEGs (left side of the heat map).

First, we compared the expression profile of patients with breast cancer before and after NAC. It was shown that the number of differentially expressed genes in the tumor before and after treatment was 378 (354 upregulated and 24 downregulated). A heat map of DEGs in the tumors of patients with breast cancer is shown in Figure 1.

Next, the top 10 upregulated and downregulated DEGs in the tumor before and after preoperative chemotherapy were identified; the results are presented in Table 1.

In addition, the top 10 signaling pathways with changes in activity in the general group under the influence of NAC were identified (*p*-value < 0.000291). These included pathways associated with *orexin receptors; **spinal cord injury; myometrial relaxation and contraction;** the development of the ureteric collection system; **hair follicle development*****, *cytodifferentiation*, *part 3 of 3; hypertrophy; IL1 and megakaryocytes in obesity; the galanin receptor; serotonin transporter activity; and*** *G alpha (s) signaling events.* It is important to note that 80% of the above signaling pathways (highlighted in bold) were 100% represented by the genes from the upregulated group.

### 2.2. Comparison of the Expression Profiles of Patients with Breast Cancer before and after Preoperative Chemotherapy Depending on the Status of Hematogenous Metastasis

**Figure 2:**
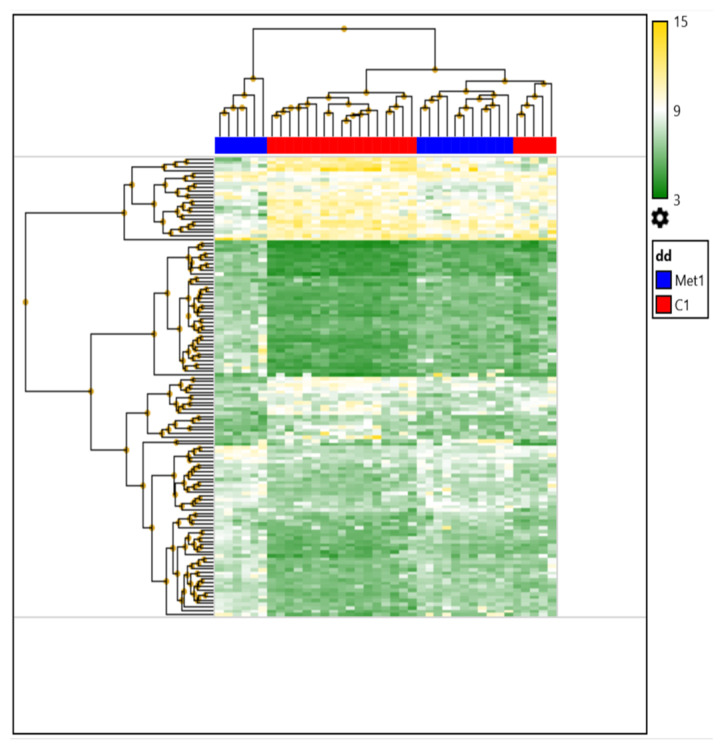
Heat map of DEG in tumors of patients with breast cancer before neoadjuvant chemotherapy, depending on the status of hematogenous metastasis.

A comparison of the expression profiles of patients with breast cancer before NAC was conducted depending on the status of distant metastasis. It was shown that the number of differentially expressed genes in the tumors before treatment in patients with and without hematogenous metastasis was 132 (38 downregulated and 94 upregulated) (Figure 2).

Next, we identified the top 10 upregulated and downregulated DEGs in the tumors before preoperative chemotherapy depending on the status of hematogenous metastasis; the results are presented in Table 2.

In an attempt to assess the changes in the expression profiles of breast cancer patients before NAC depending on the presence/absence of distant metastasis, the top 10 signaling pathways were identified. The activity of the following pathways varied depending on the status of hematogenous metastasis (*p*-value < 0.03): pathways associated with focal adhesion; *PI3K-Akt-mTOR signaling*, *MAPK signaling*, *prostaglandin synthesis and regulation*, *PI3K-Akt signaling*, *circadian rhythm genes*, *adipogenesis*, ***integrated breast cancer***, *eicosanoid synthesis*, ***calcium regulation in cardiac cells***, ***and an overview of proinflammatory and profibrotic mediators.*** It is important to note that 30% of these signaling pathways (highlighted in bold) were 100% represented by downregulated genes.

**Figure 3:**
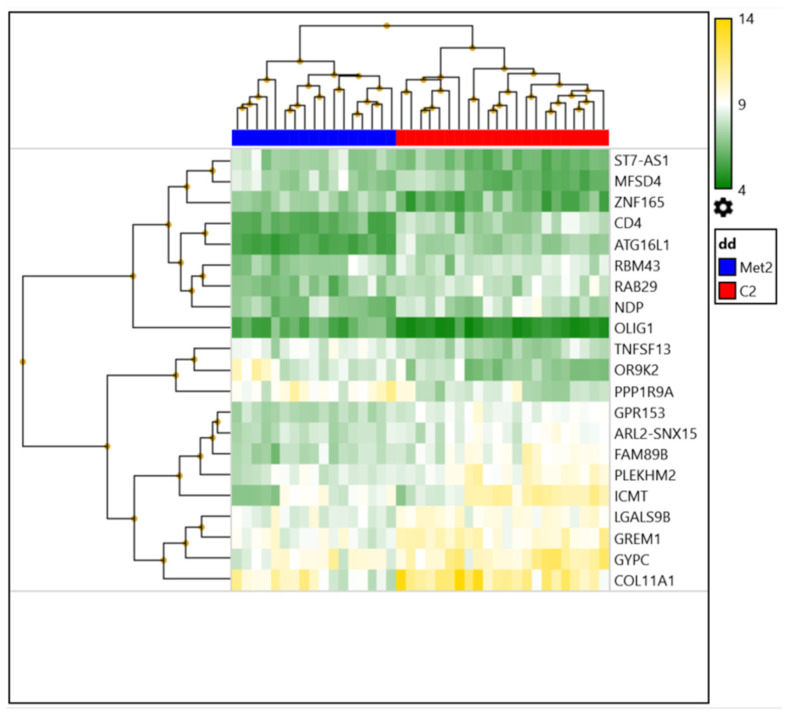
Heat map of DEG in tumors of patients with breast cancer after neoadjuvant chemotherapy, depending on the status of hematogenous metastasis.

For the assessment of changes in the expression profiles of patients with breast cancer after NAC, gene expression was compared in the residual tumors of patients with and without subsequent metastases. It was shown that the residual tumors of patients with different statuses of hematogenous metastasis differed in 21 DEGs (seven upregulated and fourteen downregulated) (Figure 3, Table 3).

In the heat map, the patients are distinctly clustered into those with distant metastasis (blue color) and those without (red color). The map shows that the screening was quite successful, and further validation may reveal genes that can be used as a panel to predict the occurrence of hematogenous metastasis in patients with breast cancer.

In addition, when assessing changes in the expression profiles of breast cancer patients after NAC in the presence or absence of distant metastasis, we identified the top 10 signaling pathways with different activities (*p*-value < 0.03), which included pathways associated with ***cytokines and the inflammatory response*, *acquired partial lipodystrophy/Barraquer–Simons syndrome*, *progeria-associated lipodystrophy***, *perturbations to host-cell autophagy induced by SARS-CoV-2 proteins*, ***autophagy***, ***LDLRAD4*, *the pathogenesis of SARS-CoV-2 mediated by the nsp9-nsp10 complex*, *the host–pathogen interaction of human coronaviruses and autophagy*, *the GDNF/RET signaling axis*, *and the selective expression of chemokine receptors during T-cell polarization.*** It is important to note that 90% of these pathways (highlighted in bold) were 100% represented by downregulated genes.

### 2.3. Validation of the Association of Genes with Hematogenous Metastasis in Patients with Breast Cancer

Validation was carried out for the upregulated and downregulated DEGs in the tumors of BC patients after neoadjuvant chemotherapy, in the presence or absence of hematogenous metastasis, by calculating metastatic-free survival (MFS) depending on the expression levels of the 21 genes considered.

**Figure 4:**
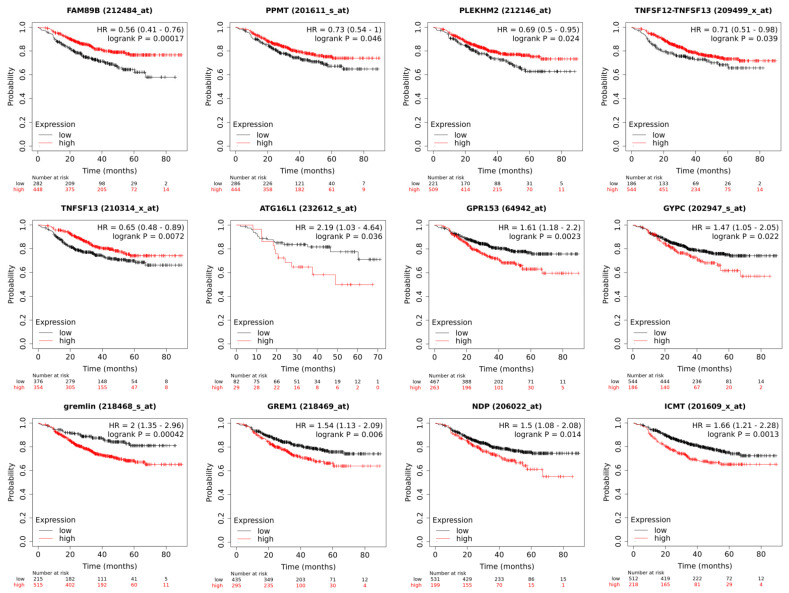
Non-metastatic survival of patients with breast cancer depending on the level of gene expression 232612_s_at (*ATG16L1*), 212484_at (*FAM89B*), 212146_at (*PLEKHM2*), 202947_s_at (*GYPC*), 218468_s_at (*GREM1*)/218469_at (*GREM1*), 206022_at (*NDP*), 201609_x_at (*ICMT*)/201611_s_at (*PPMT*), 64942_at (*GPR153*), 209499_x_at (*TNFSF12–TNFSF13*)/210314_x_at (*TNFSF13*)/223501_at (*TNFSF13*).

To complete this, data on the level of expression of the above genes and the survival rate of patients with breast cancer were assessed using the KM Plotter software (the KM-plotter is headquartered at the Semmelweis University in Budapest; Recent updates 23 August 2022) (https://kmplot.com/analysis/, accessed on 1 July 2022) [14]; here, we only selected samples from patients treated with NAC. Of the twenty-one genes, the following nine showed statistically significant changes: 212484_at (*FAM89B*), 212146_at (*PLEKHM2*), 201611_s_at (*PPMT*)/201609_x_at (*ICMT*), 64942_at (*GPR153*), 209499_x_at (*TNFSF12–TNFSF13*)/210314_x_at (*TNFSF13*)/223501_at (*TNFSF13*), 232612_s_at (*ATG16L1*), 202947_s_at (*GYPC*), and 218468_s_at (*GREM1*)/218469_at (*GREM1*), 206022_at (*NDP*) (Figure 4).

Figure 4 shows the MFS of patients with breast cancer depending on the level of expression of each of these genes.

In accordance with the data from the KM Plotter software, we found that low levels of expression of 212484_at (*FAM89B*), 212146_at (*PLEKHM2*), 201611_s_at (*PPMT*), 64942_at (*GPR153*), and 209499_x_at (*TNFSF12–TNFSF13*)/210314_x_at (*TNFSF13*)/223501_at (*TNFSF13*) were associated with an unfavorable outcome. In contrast to the data from the KM Plotter software, in our study, low levels of 232612_s_at (*ATG16L1*), 202947_s_at (*GYPC*), 218468_s_at (*GREM1*)/218469_at (*GREM1*), 206022_at (*NDP*), and 201609_x_at (*ICMT*) and high levels of 209499_x_at (*TNFSF12–TNFSF13*), 210314_x_at (*TNFSF13*), and 223501_at (*TNFSF13*) were associated with a favorable outcome.

**Figure 5:**
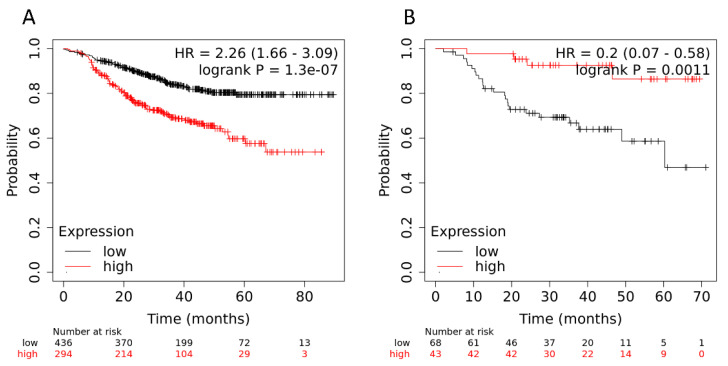
Metastatic-free survival of patients with breast cancer depending on the signature of gene expression (**A**) (64942_at (*GPR153*), 202947_s_at (*GYPC*), 218468_s_at (*GREM1*)/218469_at (*GREM1*), 206022_at (*NDP*), 201609_x_at (*ICMT*)) and (**B**) 212484_at (*FAM89B*), 212146_at (*PLEKHM2*), 201611_s_at (*PPMT*), 209499_x_at (*TNFSF12–TNFSF13*)/210314_x_at (*TNFSF13*)/223501_at (*TNFSF13*), 232612_s_at (*ATG16L1*).

We grouped these genes into two signatures: The first included five genes for which high expression was an unfavorable trait (64942_at (*GPR153*), 202947_s_at (*GYPC*), 218468_s_at (*GREM1*)/218469_at (*GREM1*), 206022_at (*NDP*), and 201609_x_at (*ICMT*)), while the second included five genes for which high expression was a favorable trait (212484_at (*FAM89B*), 212146_at (*PLEKHM2*), 201611_s_at (*PPMT*), 209499_x_at (*TNFSF12–TNFSF13*)/210314_x_at (*TNFSF13*)/223501_at (*TNFSF13*)), and 232612_s_at (*ATG16L1*) (Figure 5). These signatures showed significant predictive value, with HR = 2.26 (1.66–3.09) at *p* value = 1.3 × 10^−7^ and HR = 0.2 (0.07–0.58) at *p* value = 0.0011, respectively. Figure 5 shows the MFS of patients with breast cancer depending on the two expression patterns. It should be taken into account that when the data from the KM Plotter software included the surgical material of patients who did not receive NAC, the signatures showed statistical significance in relation to MFS.

In summary, it was shown that the expression levels of nine DEGs (subdivided into two five-gene signatures) in the tumors of BC patients after NAC—taking into account the status of hematogenous metastasis—were significantly correlated with the MFS of the patients. Thus, these signatures may be considered as two panels of markers that can be used to predict the occurrence of hematogenous metastasis in breast cancer patients with residual tumors after NAC.

## 3. Discussion

By comparing the gene expression profiles of patients after preoperative chemotherapy depending on the status of hematogenous metastasis, two panels of markers were obtained to predict the occurrence of distant metastasis: 64942_at (*GPR153*), 202947_s_at (*GYPC*), 218468_s_at (*GREM1*)/218469_at (*GREM1*), 206022_at (*NDP*), and 201609_x_at (*ICMT*)) and (212484_at (*FAM89B*), 212146_at (*PLEKHM2*), 201611_s_at (*PPMT*), 209499_x_at (*TNFSF12–TNFSF13*)/210314_x_at (*TNFSF13*)/223501_at (*TNFSF13*)), and 232612_s_at (*ATG16L1*) (HR = 2.26 (1.66–3.09) with *p* value = 1.3 × 10^−7^ and HR = 0.2 (0.07–0.58) with *p* value = 0.0011, respectively).

Several studies on the relationship between the genes included in the signatures described above, and the course of breast cancer can be found in the literature. L. Liu et al. used TargetScan technology (a miRNA target prediction algorithm) to detect downstream miR-20a targets in the autophagy pathway. Several genes associated with autophagy, including *BECN1*, ***ATG16L1***, and *SQSTM1*, were identified as putative targets. According to the results of quantitative PCR, the overexpression of miR-20a significantly reduced the number of transcripts *BECN1*, *ATG16L1*, and *SQSTM1* in the MDA-MB-231 and MCF7 cell lines. Immunoblotting showed that miR-20a repressed the protein expression of BECN1, ATG16L1, and SQSTM1 in both cell cultures [12]. In our study (Table 3 and Figure 4), we also observed that a low level of ATG16L1 expression was associated with an unfavorable outcome, verifying the results reported by Liu et al. [12].

Ankush Maind and Shital Raut 2019 proposed a new approach to identify the key genes (as diagnostic and prognostic biomarkers) involved in basal-like breast cancer (BLBC) using a biclustering algorithm and a gene co-expression network (GCN). According to their results, the key gene ***GPR153*** had the highest connectedness [15].

In one study, the expression analysis of clinical breast cancer datasets revealed that the high expression of ***GREM1*** in breast cancer stroma was correlated with a poor prognosis regardless of the molecular subtype. The large majority of human breast cancer cell lines did not express *GREM1* in vitro, while breast CAFs expressed *GREM1* both in vitro and in vivo. Transforming growth factor β (TGFβ)—secreted by breast cancer cells—and inflammatory cytokines stimulated *GREM1* expression in CAFs. In addition, Grem1 abolished bone morphogenetic protein (BMP)/SMAD signaling in breast cancer cells and promoted their mesenchymal phenotype, stemness, and invasion [16].

In another study, ***GYPC*** gene expression patterns (using ONCOMINE, GENT2, and GTX2 networks) in breast cancer and patient survival datasets were analyzed using several bioinformatics tools (including Oncomine). A decrease in *GYPC* expression was significantly correlated with high patient survival [17]. This is consistent with the data we collected from the validation; however, a low level of gene expression was correlated with a low survival rate for patients in our study.

***ICMT*** encodes for a unique last-stage enzyme in the post-translational processing pathway that modifies several oncogenic proteins, including RAS. The inhibition of ICMT was shown to lead to a decrease in self-renewal/stem potential in KRAS-driven breast cancer cells. In addition, it was functionally confirmed that the signaling cascade induced by the modification of KRAS by ICMT to form the stable TAZ protein supports the ability of tumor cells to self-renew both in vitro and in vivo [18]. Our data and validation showed that low ICMT was associated with poor outcomes, which is inconsistent with the results reported by Chai et al.; however, the validation showed that low ICMT levels were associated with a favorable outcome, while 201611_s_at (PPMT) exhibited an opposite predictive value.

In one study, it was shown that the endogenous expression of ***TNFSF13*** in a group of TN breast cancer cell lines was strongly correlated with the concentrations of paclitaxel and doxorubicin. While the knockdown of *TNFSF13* enhanced the efficacy of paclitaxel in paclitaxel-insensitive MDA-MB231 cells, recombinant *TNFSF13* (recTNFSF13) reduced the sensitivity of paclitaxel-responsive HCC1806 cells to paclitaxel treatment. In addition, a Kaplan–Meier analysis showed that an increase in the expression of *TNFSF13* mRNA significantly predicted an increased risk of relapse in ER-BC patients treated with anthracyclines. Accordingly, higher levels of the *TNFSF13* protein were found in TN patients that did not respond to anthracycline-based treatments. *TNFSF13* expression is inversely associated with the activity of the Akt-mTOR pathway, which acts as a negative regulator of autophagy activity. The pharmaceutical inhibition of autophagy activity restored the therapeutic efficacy of paclitaxel in TNFSF13-treated HCC1806 cells. These data suggest that *TNFSF13* may serve as a prognostic biomarker for chemotherapy decisions in patients with TN breast cancer [19]. This is consistent with our data, where a high level of *TNFSF13* gene expression in tumors after NAC was associated with an unfavorable outcome in patients treated with doxorubicin and taxanes. However, our validation with the KM Plotter software showed that low *TNFSF13* levels were associated with a poor outcome. This discrepancy is still difficult to explain; however, it may be due to the duality of autophagy.

In the work “Analysis of Genomic Alterations Associated with Recurrence in Early Stage Her2-Positive Breast Cancer”, gene expression was compared in the primary tumors of patients with recurrence and nonrecurrence to gain insight into the biology of high-risk HER2-positive early breast cancer. Patients who underwent curative resection and received adjuvant trastuzumab for HER2-positive early breast cancer were evaluated, and gene expression analyses were performed using NanoString Technologies’ nCounter Breast Cancer 360 Panel. In addition, PAM50 intrinsic subtypes and breast cancer signatures, including a tumor inflammation signature (TIS), were evaluated. Of the 247 patients, 28 (11.3%) presented with recurrence at a median follow-up of 54.2 months. Patients exhibiting pathological stage III, tumor size > 5 cm, axillary lymph node metastases, and hormone receptor negativity were more frequently observed in the recurrent group when compared with the nonrecurrent group. In patients with recurrence, seven genes were significantly upregulated, including *WNT11*, *HAPLN1*, *FGF10*, *BBOX1*, *CXADR*, ***NDP***, and *EREG*, and two genes were downregulated, including *CXCL9* and *GNLY*. The TIS score was significantly lower in patients with recurrence compared to the controls without recurrence. These findings suggest that the activation of oncogenic signaling pathways related to cell proliferation, adhesion, cancer stemness, and a noninflammatory tumor microenvironment are associated with the risk of recurrence in early-stage HER2-positive breast cancer [20].

## 4. Materials and Methods

***Patients and Treatment.*** This study included 39 patients with T1-3N0-2M0 breast cancer (stages IIA–IIIB) of the luminal B HER2− subtype (ER+, PR+/−, Ki67 > 30%), with a morphologically verified diagnosis, aged 25–68 years (average age 48.7 ± 0.3). Lymphogenous metastases were detected in some patients during the initial diagnosis. All patients received 6–8 courses of systemic NAC (AC, CAX, CP, ACT, AT, and Taxotere in mono mode). The visualization of the primary breast damage was performed using mammography and ultrasound. All patients underwent surgery (radical/subcutaneous mastectomy, radical resection, sectoral resection with axillary lymphadenectomy, or another type of organ-preserving surgery) followed by hormonal therapy and radiation therapy (in the presence of lymphatic metastases). Clinical responses were classified by the WHO grading system and defined as complete regression (PR, pCR), partial regression (PR), stabilization (ST), or progression (P).

This study included only patients who did not achieve complete morphological regression with an RCB greater than 1. Distant hematogenous metastasis was considered for at least five years after the end of treatment. This was the development of metastases in secondary organs, excluding lymphogenous metastasis.

Table 4 presents the clinical and morphological parameters of the patients included in the study.

***The materials*** used were paired samples of biopsy material before treatment and surgical material from each of the patients. The design of the study required a full transcriptome analysis of the breast tumor before and after preoperative chemotherapy. The tumor biopsy material was taken before treatment using a biopsy gun under ultrasound guidance. RNA was isolated from the paired samples using an RNeasy Plus Mini Kit (Qiagen, Hilden, Germany) according to the manufacturer’s instructions.

### 4.1. IHC Molecular Subtype Analysis

Data on the molecular subtype of the tumor were obtained from pathologist reports. Estrogen and progesterone receptor expression testing was performed according to the procedure described in [21], while Her2neu expression testing was carried out according to the method outlined in [22].

Luminal A tumors were ER+, PR+, HER2−, and Ki67 < 20%; Luminal B tumors were ER+, PR+, HER2−, and Ki67 > 20% or ER+, PR+, HER2+; triple-negative tumors were ER−, PR−, and HER2−; and the HER2 subtype were ER−, PR−, and HER2+.

The study included only patients with luminal B HER2− subtype. Testing was carried out at the stage before treatment and after NAC. A change in the molecular subtype during treatment (no more than 7.0%) was the basis for exclusion from the study. Thus, the tumors of patients before and after NAC did not differ in molecular subtypes.

### 4.2. Microarray Analysis

A full transcriptome microarray analysis was performed using the human Clariom™ S Assay platform (Affymetrix, USA) at The Core Facility of Medical Genomics, Tomsk NRMC.

Statistical analysis. The Transcriptome Analysis Console (TAC) software (version 4.0) was used to process the results of the microarray (DEG analysis, including the construction of heat maps and determination of signaling pathways).

### 4.3. DEG Identification

The microarray analysis provided raw expression data, from which the Transcriptome Analysis Console (TAC) software (version 4.0) was used to identify DEGs and correct for the false discovery rate (FDR) with multiple comparisons (FDR *p* value < 0.05). The DEG threshold was set to *p*-value < 0.05 (fold-change: >2 or <−2). An ANOVA analysis adjusted by eBayes was used to identify DEGs. The eBayes analysis corrects the variance of the ANOVA analysis with an empirical Bayes approach that uses the information from all the probe sets to yield an improved estimate for the variance. A probe set was considered to be expressed if 50% of the samples in the dataset had DABG (detected above background) values below the DABG threshold. The DABG threshold was set to 0.05, while the pos/neg AUC threshold was set to >0.7.

### 4.4. Identification of Significant Signaling Pathways

The TAC software (version 4.0) was used to determine all statistically significant signaling pathways (*p* value < 0.05 and FDR < 0.05).

### 4.5. Study Design

**Figure 6:**
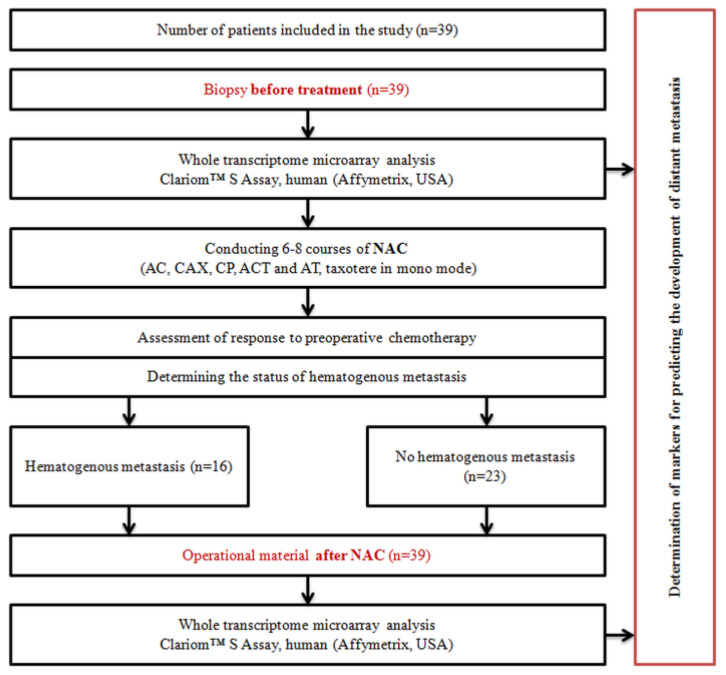
Study design.

A schematic showing the design of the study is shown in Figure 6.

## 5. Conclusions

In the present work, we compared the expression profiles of patients with breast cancer before and after preoperative chemotherapy. This made it possible to detect DEGs in tumors before and after treatment to determine the top 10 upregulated and downregulated DEGs among 378 genes and establish the signaling pathways whose activity changed under the NAC in the general group. The expression profile of breast cancer patients before and after NAC was compared depending on the status of hematogenous metastasis. According to the results, the number of DEGs after NAC in patients with breast cancer decreased by more than six times (132 DEGs and 21 DEGs in the tumors of patients before and after treatment, with and without hematogenous metastasis, respectively). Finally, the top 10 signaling pathways were found, the activity of which varied depending on the status of hematogenous metastasis before and after NAC.

We evaluated the association of DEGs with hematogenous metastasis in patients with breast cancer by assessing MFS depending on the expression levels of the 21 genes considered. It was shown that MFS was significantly associated with the expression levels of nine genes: *212484_at (FAM89B)*, *212146_at (PLEKHM2)*, *201611_s_at (PPMT)/201609_x_at (ICMT)*, *64942_at (GPR153)*, *209499_x_at (TNFSF12–TNFSF13)/210314_x_at (TNFSF13)/223501_at (TNFSF13)*, *232612_s_at (ATG16L1)*, *202947_s_at (GYPC)*, *218468_s_at (GREM1)/218469_at (GREM1)*, and *206022_at (NDP).* Additionally, we tested the association between MFS and the expression patterns of these nine genes. It was shown that the expression level of the nine DEGs in the tumors of patients with breast cancer after NAC was significantly correlated with MFS when taking into account the status of hematogenous metastasis.

The data obtained are promising for further research (prospective validation) in terms of the possibility of using them as a panel of markers for predicting the occurrence of hematogenous metastasis in patients with luminal breast cancer.

## Figures and Tables

**Table 1:** Top-10 upregulated and downregulated DEGs in the tumor BC patients before and after NAC chemotherapy.

Upregulated DEGs	Exp. Levels (log2)		Genomic Location
	Before NAC	After NAC	
*DUSP1 (Dual Specificity Phosphatase 1)*	5.38	7.94	5q35.1
*DCDC2 (Doublecortin Domain Containing 2)*	7.1	9.2	6p22.3
*FOS (Fos Proto-oncogene*, *AP-1 Transcription Factor Subunit)*	7.4	9.1	14q24.3
*NR4A3 (Nuclear Receptor Subfamily 4 Group A Member 3)*	6.38	8.03	9q22
*CYR61 (Cellular Communication Network Factor 1)*	7.56	9.06	1p22.3
*PTGS2 (Prostaglandin-Endoperoxide Synthase 2)*	6.36	7.78	1q31.1
*MGARP (Mitochondria Localized Glutamic Acid Rich Protein)*	6	7.41	4q31.1
*MGMT (O-6-Methylguanine-DNA Methyltransferase)*	8.94	10.25	10q26.3
*ENDOD1 (Endonuclease Domain Containing 1)*	7.15	8.3	11q21
*SHH (Sonic Hedgehog Signaling Molecule)*	4.9	5.95	7q36.3
**Downregulated DEGs**			
*H2AFX (H2A.X Variant Histone)*	10.59	8.38	11q23.3
*HIST1H2BL (H2B Clustered Histone 13)*	10.47	8.45	6p22.1
*MKI67 (Marker Of Proliferation Ki-67)*	10.47	8.72	10q26.2
*CENPF (Centromere Protein F)*	9.63	8.09	1q41
*UBE2C (Ubiquitin Conjugating Enzyme E2 C)*	10.66	9.13	20q13.12
*RARA (Retinoic Acid Receptor Alpha)*	10.34	8.89	17q21.2
*BOP1 (BOP1 Ribosomal Biogenesis Factor)*	9.5	8.22	8q24.3
*TROAP (Trophinin Associated Protein)*	7.33	6.16	12q13.12
*OR10Q1 (Olfactory Receptor Family 10 Subfamily Q Member 1)*	6.48	5.45	11q12.1
*NKAIN1 (Sodium/Potassium Transporting ATPase Interacting 1)*	8	6.98	1p35.2

**Table 2:** Top-10 upregulated and downregulated DEGs in the tumor BC patients before and after NAC chemotherapy, depending on the absence/presence of hematogenous metastasis.

Upregulated DEGs	Exp. Levels (log2)		Genomic Location
	No Mts	Yes Mts	
*VWC2L (Von Willebrand Factor C Domain Containing 2 Like)*	5.13	6.9	2q34-q35
*MTA3 (Metastasis Associated 1 Family Member 3)*	5.21	6.67	2p21
*CHN2 (Chimerin 2)*	6.23	7.62	7p14.3
*GRXCR2 (Glutaredoxin And Cysteine Rich Domain Containing 2)*	4.79	6.25	5q32
*SPAG5 (Sperm Associated Antigen 5)*	5.19	6.59	17q11.2
*IL19 (Interleukin 19)*	5.86	8.08	1q32.1
*RAB41 (Member RAS Oncogene Family)*	4.52	6.01	Xq13.1
*ABCB4 (ATP Binding Cassette Subfamily B Member 4)*	5.7	7.1	7q21.12
*HIST1H2BB (H2B Clustered Histone 3)*	6.66	8.67	6p22.2
*HIST1H2BI (H2B Clustered Histone 10)*	5.36	7.39	6p22.2
**Downregulated DEGs**			
*MGMT (O-6-Methylguanine-DNA Methyltransferase)*	10.01	8.26	10q26.3
*PTGIS (Prostaglandin I2 Synthase)*	9.82	8.1	20q13.13
*INIP (INTS3 And NABP Interacting Protein)*	10.78	8.8	9q32
*HLA-E (Major Histocompatibility Complex*, *Class I*, *E)*	11.21	8.93	6p22.1
*LRIG3 (Leucine-Rich Repeats and Immunoglobulin-Like Domains 3)*	8.71	6.65	12q14.1
*EHD2 (EH Domain Containing 2)*	11.67	8.34	19q13.33
*EPPK1 (Epiplakin 1)*	11.07	9.17	8q24.3
*CITED2 (Cbp/P300 Interacting Transactivator With Glu/Asp Rich Carboxy-Terminal Domain 2)*	11.05	8.95	6q24.1
*MCAT (Malonyl-CoA-Acyl Carrier Protein Transacylase)*	9.38	7.43	22q13.2
*REEP6 (Receptor Accessory Protein 6)*	12.13	8.83	19p13.3

**Table 3:** Upregulated and downregulated DEG in tumors of patients with breast cancer after neoadjuvant chemotherapy, depending on the absence/presence of hematogenous metastasis.

Upregulated DEGs	Exp. Levels (log2)		Genomic Location
	No Mts	Yes Mts	
*OLIG1 (Oligodendrocyte Transcription Factor 1)*	4.69	5.98	21q22.11
*TNFSF13 (TNF Superfamily Member 13)*	7.53	8.67	17p13.1
*ST7-AS1 (ST7 Antisense RNA 1)*	6.24	7.25	7q31.2
*ZNF165 (Zinc Finger Protein 165)*	6.01	7.11	6p22.1
*PPP1R9A (Protein Phosphatase 1 Regulatory Subunit 9A)*	7.93	9.26	7q21.3
*OR9K2 (Olfactory Receptor Family 9 Subfamily K Member 2)*	7	8.3	12q13.2
*MFSD4 (Major Facilitator Superfamily Domain Containing 4A)*	5.99	7.31	1q32.1
**Downregulated DEGs**			
*ATG16L1 (Autophagy Related 16 Like 1)*	7.19	5.46	2q37.1
*CD4 (CD4 Molecule)*	7.61	5.76	12p13.31
*GPR153 (G Protein-Coupled Receptor 153)*	8.74	7.51	1p36.31
*FAM89B (Family With Sequence Similarity 89 Member B)*	9.14	7.7	11q13.1
*RAB29 (Member RAS Oncogene Family)*	7.87	6.79	1q32.1
*ARL2-SNX15 (Readthrough (NMD Candidate))*	8.85	7.8	11q13.1
*PLEKHM2 (Pleckstrin Homology And Domain Containing M2)*	9.37	8.32	1p36.21
*COL11A1 (Collagen Type XI Alpha 1 Chain)*	11.11	9.36	1p21.1
*GYPC (Glycophorin C (Gerbich Blood Group))*	10.44	9.39	2q14.3
*GREM1 (Gremlin 1*, *DAN Family BMP Antagonist)*	9.97	8.86	15q13.3
*LGALS9B (Galectin 9B)*	9.56	8.45	17p11.2
*NDP (Norrin Cystine Knot Growth Factor NDP)*	7.8	6.67	Xp11.3
*ICMT (PPMT) (Isoprenylcysteine Carboxyl Methyltransferase)*	10.54	8.56	1p36.31
*RBM43 (RNA Binding Motif Protein 43)*	8.1	6.99	2q23.3

**Table 4:** Clinical and morphological parameters of the patients with breast cancer.

Clinical and Morphological Parameter		The Number of Patients, abs.n. (%)
Menstrual status	Premenopause	22 (56.4%)
	Postmenopause	17 (43.6%)
Histological type	Invasive ductal carcinoma	34 (87.2%)
	Invasive lobular carcinoma	2 (5.1%)
	Other types	3 (7.7%)
Tumor size	T_1-2_	36 (92.3%)
	T_3-4_	3 (7.7%)
Lymphogenous metastasis	N_0_	16 (41.0%)
	N_1-2_	23 (58.9%)
Hematogenous metastasis	Yes	16 (41.0%)
	No	23 (59.0%)
NAC regimen	CAX	8 (20.5%)
	АС	18 (46.1%)
	Taxotere in mono	6 (15.4%)
	АТ/АСТ	3 (7.7%)
	CP	4 (10.3%)
NAC effect	Progression and stabilization	12 (30.8%)
	Partial regression	27 (69.2%)
Median observation	64 [14; 144]	39 (100%)

## Data Availability

Database registration certificate RU 2022620758 4 June 2022, Ibragimova, M.K., Tsyganov, M.M., Garbukov E.Yu., Zdereva E.A., Usynin E.A., Litviakov, N.V. Transcriptomic profile database of breast cancer patients with hematogenous metastasis.
